# Normal saline resuscitation worsens lactic acidosis in experimental sepsis

**DOI:** 10.1186/cc10860

**Published:** 2012-03-20

**Authors:** F Zhou, ME Cove, ZY Peng, J Bishop, K Singbartl, JA Kellum

**Affiliations:** 1 University of Pittsburgh Medical School, Pittsburgh, PA, USA

## Introduction

Infusing large volumes of 0.9% sodium chloride (saline) causes hyperchloremic acidosis. The clinical relevance of this effect remains contentious and saline is still the most commonly used resuscitation fluid in the US. However, a recent trial showed that saline or albumin in saline increased mortality in children with malarial sepsis, compared to no fluid [1]. Infusion of these fluids may have perpetuated the underlying metabolic acidosis sepsis, causing cardiovascular collapse and death. In this study, we investigated the effect of saline versus a balanced crystalloid (plasmalyte) in a cecal ligation and puncture (CLP) model of sepsis. We hypothesized that saline resuscitation would increase acidosis and worsen hemodynamics, compared to resuscitation using a balanced crystalloid.

## Methods

Fifty adult male Sprague-Dawley rats were subjected to CLP (25% cecum length, two punctures with a 25-gauge needle). Eighteen hours later, they were randomly assigned to receive either 30 ml/kg saline (*n *= 25) or plasmalyte (*n *= 25) over 4 hours. Arterial blood gases, serum creatinine, urea, and lactate were measured at baseline, 18 hours after CLP (before resuscitation), after resuscitation, and 24 hours after resuscitation. Blood pressure and pulse rate were measured during fluid infusion.

## Results

Saline-treated animals developed significantly higher levels of serum chloride (111 mmol/l vs. 102 mmol/l, *P *< 0.0001) and lower pH (7.35 v. 7.44, *P *< 0.01) compared to plasmalyte. In addition, lactate was significantly higher after fluid infusion in the saline group (4.8 mmol/l vs. 2.5 mmol/l, *P *< 0.001) compared to plasmalyte, despite being similar before infusion (2.61 vs. 2.39, *P *> 0.05). However, neither mean arterial blood pressure (83 mmHg vs. 91 mmHg, *P *> 0.10) nor heart rate (310 vs. 299, *P *> 0.10) differed between the two groups.

## Conclusion

Saline infusion worsens lactic acidosis, despite similar blood pressure, when compared to plasmalyte. The mechanisms responsible for this effect are unclear. However, deoxygenated hemoglobin readily binds hydrogen ions, forming HbH^+^, which is stabilized in the presence of chloride [2]. Consequently, the oxygen affinity for hemoglobin is reduced, which could impair oxygen delivery, perpetuating the lactic acidosis. Further study is needed to better understand the mechanisms of this effect and their clinical relevance.
